# Conservative management of a spontaneous retroperitoneal hematoma in the third trimester: a rare case report

**DOI:** 10.1186/s12884-026-08873-0

**Published:** 2026-02-25

**Authors:** Dan Zhang, Miao Hu, Yansong Liu, Qian Zhao, Zhitao Zhang

**Affiliations:** 1 Department of Obstetrics and Gynecology, Shenyang Women’s and Children’s Hospital, No.87 Da’nan Street, Shenhe District, Shenyang, Liaoning China; 2 Shenyang Clinical Medical Research Center for Obstetrics and Gynecology, Shenyang Women’s and Children’s Hospital, No.87 Da’nan Street, Shenhe District, Shenyang, Liaoning China; 3 Department of Radiology, Shenyang Women’s and Children’s Hospital, No.87 Da’nan Street, Shenhe District, Shenyang, Liaoning China

**Keywords:** Retroperitoneal hematoma, Hemorrhagic ovarian cyst, Case report

## Abstract

**Introduction:**

Spontaneous retroperitoneal hematoma during pregnancy is an extremely rare clinical event. Although not categorized as a primary obstetric emergency, it poses significant challenges in clinical diagnosis and management.

**Case presentation:**

A 30-year-old patient at 33 + 5 weeks of gestation presented with acute abdominal and low back pain. Imaging revealed a large, multiloculated cystic mass posterior to the cervix with internal hemorrhagic signals, which was initially misdiagnosed as a hemorrhagic ovarian cyst. Under ultrasound guidance, culdocentesis was performed, draining 85 mL of non-clotting blood, leading to subsequent symptom relief. Serial follow-up demonstrated overall gradual shrinkage of the mass, with a transient postoperative increase at one week, followed by continued shrinkage. An elective cesarean section was performed at 39 + 1 weeks. Intraoperative exploration identified normal bilateral ovaries, with no evidence of cysts or adhesions in the pelvic cavity. Postpartum MRI confirmed a residual mass with mixed signals in the original location, leading to a definitive diagnosis of retroperitoneal hematoma. Subsequent imaging showed near-complete resolution of the hematoma, with only organized tissue remaining. This case culminated in a successful full-term delivery with favorable outcomes for both the mother and the neonate.

**Conclusion:**

Spontaneous retroperitoneal hematoma should be considered in the differential diagnosis for pregnant women presenting with acute abdominal pain or back pain, particularly when associated with an adnexal mass. Furthermore, the favorable natural course of the hematoma observed in this stable patient provides valuable insights into the potential for conservative management of this condition.

## Introduction

Acute abdominal pain during pregnancy presents a complex diagnostic challenge with direct implications for maternal and fetal safety. While complications of ovarian cysts are a common differential diagnosis, spontaneous retroperitoneal hematoma (SRH) represents a rare yet potentially life-threatening condition. SRH is defined as bleeding into the retroperitoneal space in the absence of trauma or iatrogenic causes. Its deep anatomical location and non-specific clinical presentation often lead to diagnostic difficulties and its frequent oversight in obstetric practice [1, 2]. Although SRH is most commonly associated with anticoagulant or antiplatelet therapy [1], its occurrence in pregnant women without such identifiable predisposing factors is exceedingly rare [3]. This paper reports a case of idiopathic SRH in the third trimester that was initially misdiagnosed as a hemorrhagic ovarian cyst. The unique clinical course of this patient suggests that, in the context of hemodynamic stability, the disease may follow a clinical trajectory distinct from conventional aggressive intervention. This case aims to highlight the importance of considering SRH in the differential diagnosis during pregnancy and to underscore the feasibility of individualized assessment and conservative management in selected scenarios, thereby offering a fresh perspective on the complete clinical spectrum of this condition.

## Case presentation

**Fig. 1:**
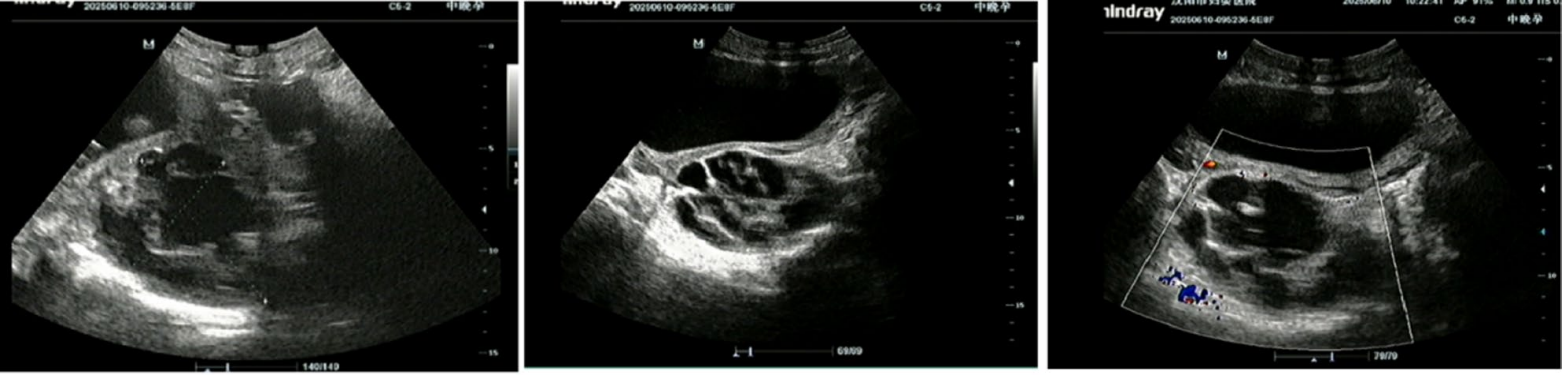
Transvaginal ultrasound on admission at 33 + 5 weeks of gestation. A cystic mass (87 mm × 68 mm × 67 mm) with internal septations is seen posterior to the cervix

**Fig. 2:**
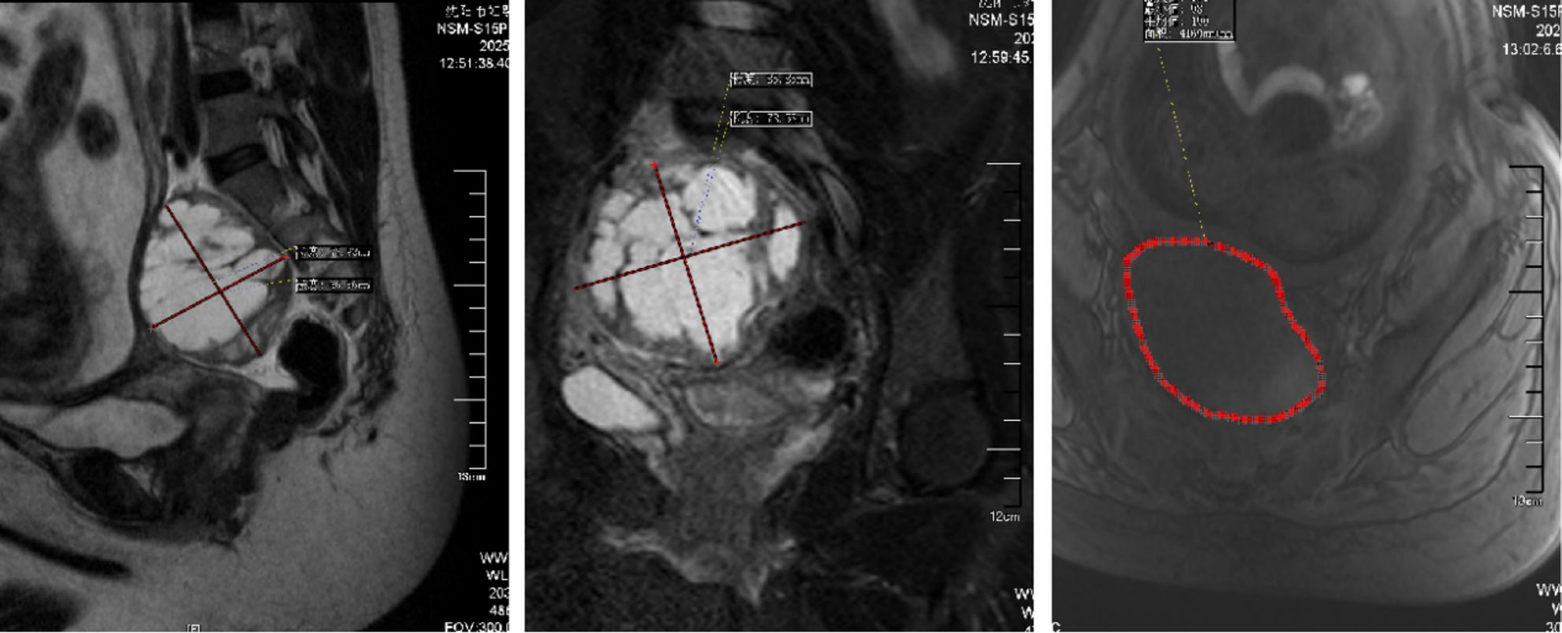
MRI on admission at 33 + 5 weeks of gestation. A cystic mass (85 mm × 73 mm × 66 mm) with mixed T1/T2 signals and septations is observed behind the cervix, suggestive of hemorrhage

A 30-year-old woman, gravida 2 para 0, was admitted to our hospital at 33 + 5 weeks of gestation due to a three day history of lower back pain and abdominal pain. The patient reported no identifiable precipitating factors prior to symptom onset. The pain was exacerbated by positional changes, required the patient to maintain a passive posture, radiated to the lower back, and was accompanied by nausea and vomiting. On physical examination, the patient’s heart rate was 70 beats/min, and blood pressure was 100/60 mmHg. Occasional uterine contractions were palpated. The abdomen was soft, without tenderness or rebound pain. Costovertebral angle tenderness was negative. Gynecological examination revealed a posterior vaginal fornix with high tension and significant tenderness. On ultrasound, A a complex cystic mass measuring 87 mm × 68 mm × 67 mm was observed posterior to the cervix (Fig. 1 ). It was irregular in shape, contained multiple internal septations, and showed no blood flow signal. On pelvic MRI, A cystic mass measuring 85 mm × 73 mm × 66 mm was identified behind the cervix (Fig. 2). It contained multiple septations and exhibited mixed T1/T2 signals (short T1, long T2 signals suggestive of hemorrhage), with punctate short T1 signals on the cyst wall. Laboratory Tests showed a hemoglobin of 130 g/L, normal coagulation function, and tumor markers (CA125, CA19-9, CEA) all within normal ranges.

The severity of the pain prompted an initial suspicion of ovarian cyst torsion. However, the overall clinical presentation was atypical. The ultrasound and MRI findings was more supportive of a diagnosis of a hemorrhagic ovarian cyst. To alleviate symptoms and prolong gestation, ultrasound-guided transvaginal culdocentesis was performed by an obstetrician under regional anesthesia to aspirate cystic fluid and reduce intracystic pressure. A total of 85 mL of non-clotting blood was drained. Postoperatively, the patient’s lumbar pain demonstrated marked resolution; she subsequently received tocolysis, prophylactic antibiotics(cefazolin), and symptomatic supportive treatment.

**Fig. 3:**
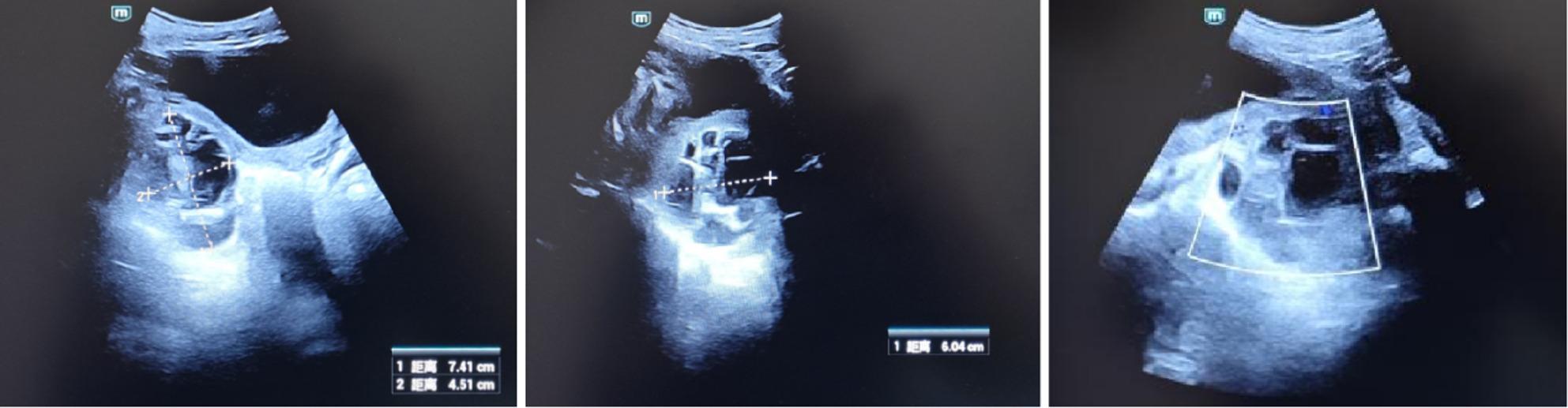
Follow-up transvaginal ultrasound 3 h post-aspiration. The cyst size has reduced to 74 × 60 × 45 mm with persistent internal septations

**Fig. 4:**
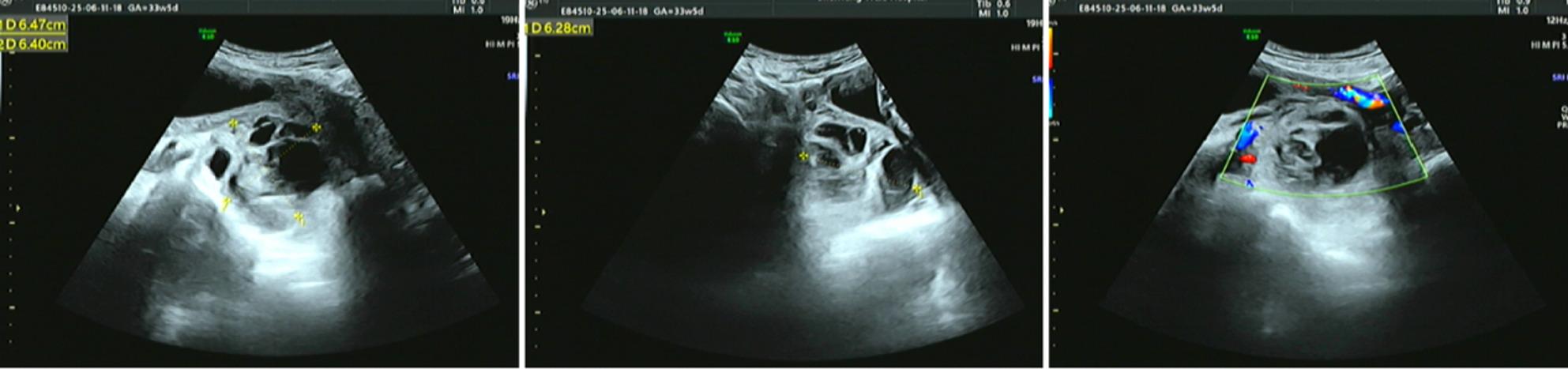
Follow-up transvaginal ultrasound on the first postoperative day. The cyst measures 65×63×63mm with no signs of active bleeding

A follow-up ultrasound 3 h post-procedure showed the cyst size reduced to 74 × 60 × 45 mm (Fig. 3), with internal septations still present. On the first postoperative day, hemoglobin decreased to 97 g/L, but the patient’s vital signs remained stable. Ultrasound examination showed the cyst size was 65 × 63 × 63 mm (Fig. 4), with no further enlargement or signs of active bleeding.

**Fig. 5:**
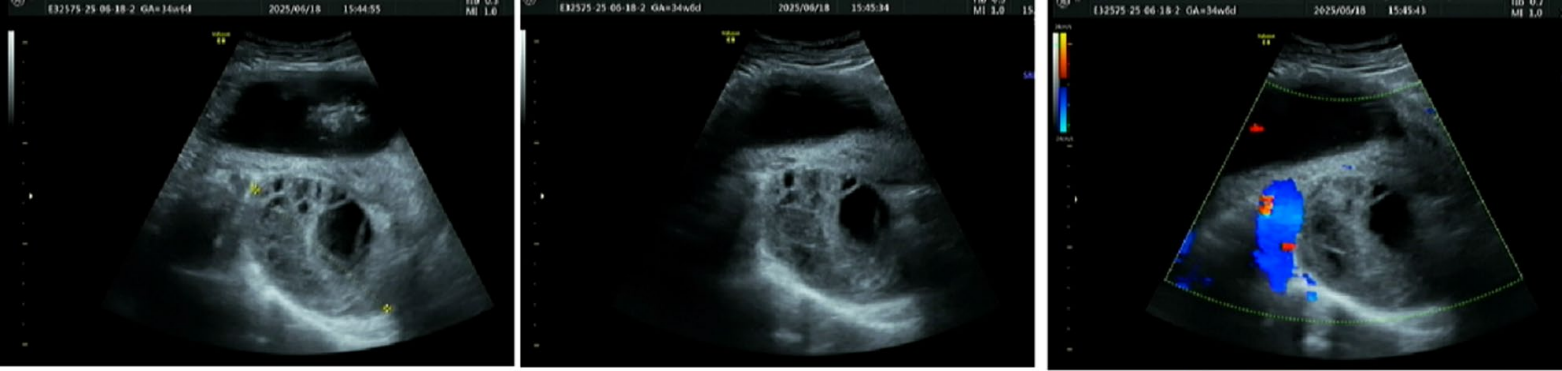
Transvaginal ultrasound at 34 + 6 weeks of gestation. The complex cystic mass posterior to the cervix has increased to 87 × 62 × 61 mm

After treatment, the patient experienced no further episodes of abdominal pain. An ultrasound at 34 + 6 weeks of gestation showed the complex cystic mass posterior to the cervix measured 87 × 62 × 61 mm (Fig. 5). By 37 + 5 weeks of gestation, the cyst was no longer detectable by ultrasound.

An elective cesarean section was performed at maternal request due to concerns over the known pelvic mass at 39 + 1 weeks, delivering a healthy female infant weighing 2630 g with Apgar scores of 10 and 10 at 1 and 5 min, respectively. Careful exploration of the pelvic cavity revealed normal bilateral ovaries in size and morphology, with smooth surfaces and no signs of rupture, cysts, or hematomas. Both fallopian tubes were normal. The surfaces of the uterus, bladder, and intestines showed no abnormalities. No indurated mass was palpated in the space posterior to the lower uterine segment and anterior to the rectum (pouch of Douglas).

**Fig. 6:**
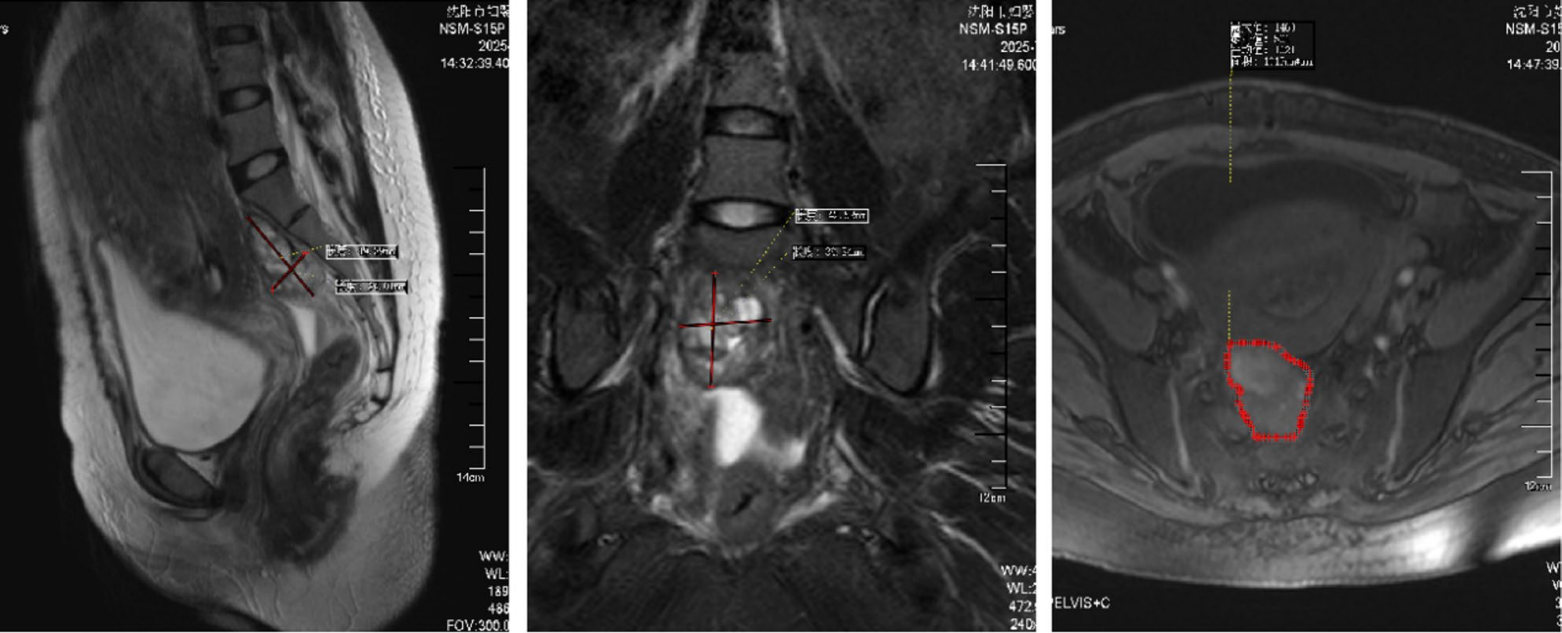
Postoperative MRI at 3 days after cesarean section. A mixed-signal mass (50 mm×26 mm×37 mm) posterior to the lower uterus, consistent with a partially organized hematoma

On postoperative follow-up MRI (3 days postnatally), a mixed-signal mass, measuring approximately 50 mm×26 mm×37 mm was observed in the retroperitoneal space posterior to the lower uterus (Fig. 6), consistent with the appearance of a partially organized hematoma. The diagnosis was revised to spontaneous retroperitoneal hematoma.

**Fig. 7:**
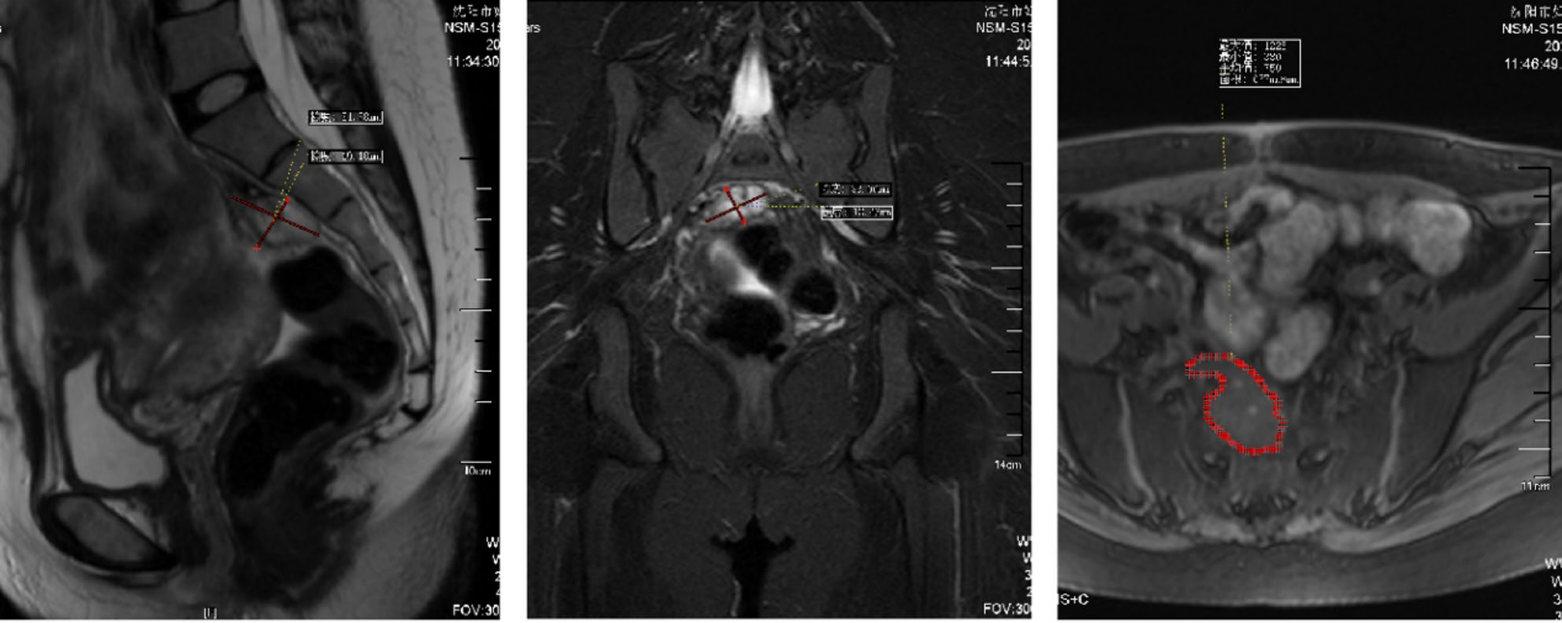
Follow-up MRI at 60 days postpartum. A mixed-signal mass (33 mm×29 mm×19 mm) located in the retroperitoneal space posterior to the lower uterus, consistent with an organized hematoma

Follow-up MRI (60 days postpartum): A mixed-signal mass, measuring approximately 33 mm×29 mm×19 mm, was seen posterior to the lower uterus (Fig. 7), consistent with the MRI appearance of an organized hematoma.

## Discussion

The initial misdiagnosis in this case stemmed from the remarkable imaging similarities among a retroperitoneal hematoma, a hemorrhagic ovarian cyst and also retroperitonal vascularized tumor. All these entities can present as a complex cystic-solid mass in the pelvic cavity, with internal septations and MRI signals characteristic of acute hemorrhage (short T1, long T2) [4, 5]. However, the direct intraoperative visualization of normal ovaries definitively ruled out an ovarian source. Combined with the subsequent MRI follow-up demonstrating the classic evolutionary process of gradual hematoma resolution, this provided a reliable basis for the revised diagnosis. The hematoma was diagnosed at 33 + 5 weeks of gestation, which is characteristic within the literature on pregnancy-associated SRH. Similar to the majority of cases reported in the mid-to-late trimester, this case also occurred during the second or third trimester. This timing may be related to physiological changes, such as increased pelvic vascular load, that progress with advancing gestational age [3, 6].The hematoma likely originated from the spontaneous rupture of the engorged pelvic venous plexus or minor capillary tears during pregnancy. The subsequent accumulation of blood in the retroperitoneal space anterior to the rectum and posterior to the uterus created a mass effect mimicking an adnexal lesion on examination.During pregnancy, SRH secondary to specific underlying diseases has well-established etiological basis. Known secondary causes include: rupture of renal angiomyolipoma [7–9], rupture of a renal artery aneurysm [10], retroperitoneal lymphangioleiomyomatosis [11], injury to the inferior phrenic artery due to severe vomiting [12], and hemorrhagic complications associated with anticoagulant therapy such as heparin [13].The idiopathic etiology and nonspecific radiological presentation of this case posed a major challenge to the initial diagnosis.

The management of this patient highlights the importance of individualized risk assessment and clinical decision-making for SRH during pregnancy. Although the initial transvaginal culdocentesis was performed under the misdiagnosis of a “hemorrhagic ovarian cyst,” this diagnostic procedure not only effectively alleviated pain and reduced intracystic pressure—potentially preventing further hematoma expansion or rupture—but also, by yielding non-clotting blood in the context of normal tumor markers, provided crucial evidence against malignancy. For a pre-term patient, this conservative strategy successfully prolonged the gestation, demonstrating the clinical rationale for opting for conservative management in cases of hemodynamically stable retroperitoneal hematoma. This case aligns with two other reported instances of successful conservative management of retroperitoneal hematomas identified post-cesarean Sects [14, 15]. It is crucial to note, however, that this approach should not be considered the standard of care for all retroperitoneal hematomas.In non-pregnant patients or those who are hemodynamically unstable, interventional radiological procedures or surgical evacuation remain necessary [1, 3, 12, 16].Even when temporary conservative management is adopted for hemorrhage due to ruptured renal tumors to prolong gestation, active surgical or interventional treatment of the underlying pathology remains necessary postpartum [8]. When maternal life is directly threatened by complications such as hemorrhage and cannot be effectively sustained, therapeutic induction of labor must be performed promptly to prioritize maternal safety [9].

Ultimately, this case achieved a favorable maternal-neonatal outcome, with maternal recovery and full-term delivery. This result is consistent with the outcomes reported in the literature for cases managed conservatively with non-malignant etiologies [8, 9]. In contrast, SRH caused by active arterial bleeding or tumor rupture is often associated with severe maternal and fetal complications, including fetal demise [6, 12]. The present case highlights the critical role of early recognition, accurate assessment (particularly of hemodynamic status and etiology), and individualized decision-making in improving the prognosis of SRH during pregnancy.

The key clinical implications from this case are twofold. When a pregnant woman presents with an acute pelvic mass accompanied by abdominal pain, SRH should be included in the differential diagnosis, even if imaging features are highly suggestive of more common entities like ovarian cysts.

As a single case report, this case illustrates that in the context of hemodynamic stability, a conservative strategy—encompassing close monitoring, symptomatic management, and potentially therapeutic culdocentesis—can effectively relieve symptoms, promote hematoma organization and resolution, and ultimately avoid exploratory laparotomy, thereby optimizing maternal and fetal outcomes. However, it must be strongly emphasized that the success of such management depends heavily on excluding active arterial bleeding and implementing intensive patient surveillance.

Several limitations of this case report must be acknowledged. This study is a single case report with a retrospective diagnosis. It is particularly important to note that the culdocentesis performed in this case was administered as a therapeutic measure under the mistaken initial diagnosis of SRH as a hemorrhagic ovarian cyst. It is neither a conventional nor a recommended treatment for suspected SRH. In fact, for SRH, this procedure carries potential risks, including infection, iatrogenic injury, and the possibility of triggering re-bleeding due to the disruption of the encapsulated hematoma by the puncture. Therefore, the symptomatic relief resulting from the puncture in this case should be regarded as an incidental benefit obtained under specific circumstances, rather than being interpreted as an active therapeutic strategy for SRH.

## Conclusion

This case illustrates that SRH is a rare but critical cause of acute abdominal pain in pregnancy, capable of mimicking the clinical and radiological presentation of ovarian cyst complications. Meticulous intraoperative exploration and serial imaging follow-up are essential for a definitive diagnosis. The management of SRH in pregnancy requires a thorough assessment of the hematoma’s characteristics, integrated with gestational age, to guide individualized treatment decisions.

## Data Availability

The data used and analysed during the current study are available from thecorresponding author on reasonable request. All data generated or analysedduring this study are included in this published article.
